# Nanomaterials Manipulate Macrophages for Rheumatoid Arthritis Treatment

**DOI:** 10.3389/fphar.2021.699245

**Published:** 2021-07-14

**Authors:** Shuang Li, Jin Su, Wei Cai, Jian-xin Liu

**Affiliations:** ^1^ Hunan Province Key Laboratory of Antibody-based Drug and Intelligent Delivery System, School of Pharmaceutical Sciences, Hunan University of Medicine, Huaihua, China; ^2^ College Pharmacy, Jiamusi University, Jiamusi, China

**Keywords:** nanomaterials, macrophages, rheumatoid arthritis, inflammation, treatment

## Abstract

Rheumatoid arthritis (RA) is a chronic, progressive, and systemic inflammatory autoimmune disease, characterized by synovial inflammation, synovial lining hyperplasia and inflammatory cell infiltration, autoantibody production, and cartilage/bone destruction. Macrophages are crucial effector cells in the pathological process of RA, which can interact with T, B, and fibroblast-like synovial cells to produce large amounts of cytokines, chemokines, digestive enzymes, prostaglandins, and reactive oxygen species to accelerate bone destruction. Therefore, the use of nanomaterials to target macrophages has far-reaching therapeutic implications for RA. A number of limitations exist in the current clinical therapy for patients with RA, including severe side effects and poor selectivity, as well as the need for frequent administration of therapeutic agents and high doses of medication. These challenges have encouraged the development of targeting drug delivery systems and their application in the treatment of RA. Recently, obvious therapeutic effects on RA were observed following the use of various types of nanomaterials to manipulate macrophages through intravenous injection (active or passive targeting), oral administration, percutaneous absorption, intraperitoneal injection, and intra-articular injection, which offers several advantages, such as high-precision targeting of the macrophages and synovial tissue of the joint. In this review, the mechanisms involved in the manipulation of macrophages by nanomaterials are analyzed, and the prospect of clinical application is also discussed. The objective of this article was to provide a reference for the ongoing research concerning the treatment of RA based on the targeting of macrophages.

## Introduction

**FIGURE 1:**
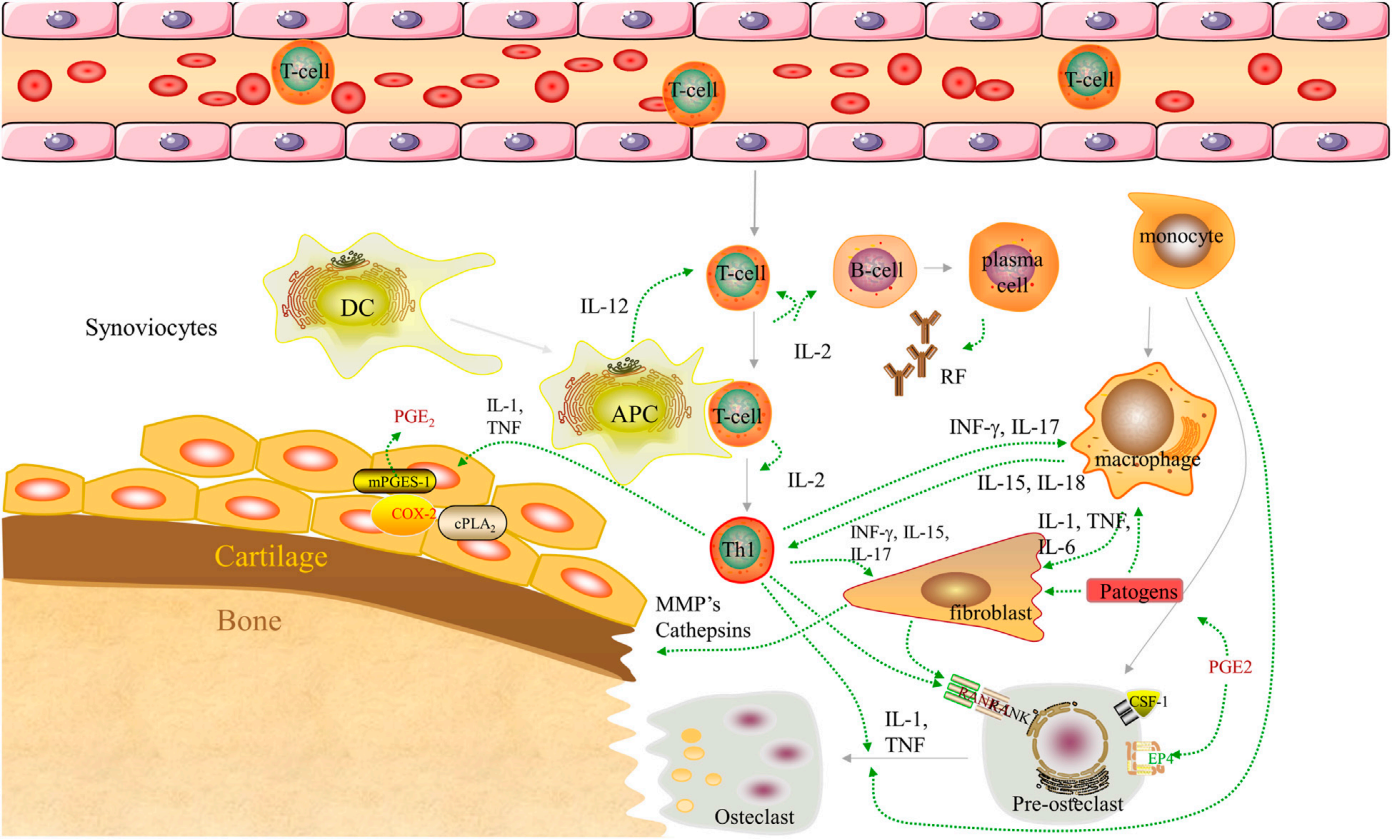
Pathogenesis of RA. Dendritic cells complexed with autoantigen to activate naive T cells. Differentiated T cells stimulate B cells to produce autoantibodies [rheumatoid factor (RF)]. B cells present autoantigens to T cells, leading to their activation. Synovial macrophages are activated by T cells to release pro-inflammatory cytokines (TNF-α, IL-1, and IL-6), driving the activation of synovial fibroblasts and inducing osteoclast production. In addition, the monocyte subpopulations of the arthritic synovium can differentiate into macrophages and osteoclasts. Collectively, these components play an important role in the destruction of bones, cartilage, and the synovium. APC, antigen-presenting cell; COX-2, cyclooxygenase-2; cPLA2, cyosolic phospholipase A2; CSF-1, colony-stimulating factor-1; DC, dendritic cell; EP4, prostaglandin E receptor subtype 4; IFN-ɣ, interferon-gamma; IL-, interleukin; MMP, matrix metalloproteinase; mPGES-1, microsomal prostaglandin E synthase-1; PGE2, prostaglandin E2; PGF2, prostaglandin F2; RA, rheumatoid arthritis; RANK, receptor activator of nuclear factor-kB; Th1, T helper 1; TNF-α, tumor necrosis factor α.

Rheumatoid arthritis (RA) is the most common inflammatory autoimmune disease and is characterized by immune cell infiltration (e.g., macrophages) and chronic inflammation in the synovium tissue. The global prevalence of RA in patients aged 5–100 years was approximately 0.24% (95% confidence interval: 0.23–0.25%). An approximately three-fold higher incidence of RA was observed in females vs. males, that is, 0.35% (0.34–0.37%) vs. 0.13% (0.12–0.13%), respectively (Cross et al., 2014). The exact etiology of RA is currently unknown. However, it has been demonstrated that a number of effector cells (e.g., macrophages, T lymphocytes, and dendritic cells), inflammatory cytokines, such as tumor necrosis factor α (TNF-α), interleukin 1 (IL-1), and interleukin 6 (IL-6), and their interactions contribute to the pathological process of RA (Figure 1) (Smolen et al., 2007; Aletaha and Smolen, 2018).

Promonocytes exist in the bloodstream and can differentiate into monocytes. Subsequently, these cells extravasate into tissues, where they further differentiate into a specific type of “resident” tissue macrophage (Maruotti et al., 2007). The number of macrophages is often increased in the synovium lining of patients with RA (Athanasou, 1995). Macrophages are important immune cells causing a sustainable chronic inflammatory response in the synovial tissue of patients with RA. Activated macrophages act through the release of pro-inflammatory cytokines (e.g., TNF-α, IL-1β, and IL-6) and inflammatory mediators (e.g., prostaglandins and chemokines), which maintain the chronic inflammation and result in pain, an increase in inflammatory cell infiltration, the formation of synovial pannus, and joint destruction (Ma and Pope, 2005). More importantly, the recruitment of macrophages to the site of inflammatory response results in persistent and amplified inflammation (Gao et al., 2021). Therefore, the targeting of macrophages is an important approach to treating inflammation and RA.

Although substantial evidence and experience with regard to the treatment of RA have been accumulated over the past few decades, the effective management of this disease remains a challenge. Currently, the major objectives of drug therapy are to alleviate the symptoms of RA and reduce the disease activity (Smolen et al., 2010). Several drugs are recommended by the European League Against Rheumatism for the management of RA. Based on the guidelines, treatment with synthetic and biological disease-modifying antirheumatic drugs (DMARDs), including conventional synthetic DMARDs [methotrexate (MTX), leflunomide, and sulfasalazine] and biological DMARDs (adalimumab, certolizumab pegol, and etanercept), should be promptly initiated following the diagnosis of RA. This highlights that DMARDs play an irreplaceable role in the pharmacological strategies for the treatment of RA. Meanwhile, glucocorticoids (GCs) can also be used as bridging therapy until conventional synthetic DMARDs produce observable effects. In China, traditional Chinese medicine (TCM) (e.g., sinomenine preparations, total glycosides of paeony capsules, and preparations from the plant *Tripterygium*) has been applied to the treatment for RA. The mechanisms and characteristics of action of TCM on RA are similar to those of DMARDs; however, TCM requires a long period of therapy to alleviate symptoms of RA (Zhang et al., 2010).

There are numerous problems in the pharmacological treatment of RA. Long-term use of nonsteroidal anti-inflammatory drugs (NSAIDs), DMARDs, and GCs has serious adverse effects on gastrointestinal, cardiovascular, liver, and kidney functions (Oray et al., 2016; Schett et al., 2016; Buttgereit, 2020). Although biological agents are associated with a rapid onset of effect, they are limited by their high cost, which leads to poor patient compliance (Dalal et al., 2019). The use of TCM compounds in clinical practice is linked to definite therapeutic effects and a low incidence of side effects. Through the compatibility of medicines, TCM compounds can increase the effectiveness of the treatment and reduce the toxicity. However, the large differences in the composition and dosage of different formulations complicate their application. The compositions are often complex and occasionally have ill-defined active ingredients; moreover, the efficacy of these compounds is not supported by robust scientific research data (Burmester and Pope, 2017; Xing et al., 2020). Therefore, it is important to overcome the disadvantages of these agents in the treatment of RA. Recently, research has shown that various nanomaterials can be used to carry anti-RA drugs by manipulating macrophages. This approach improves the solubility and bioavailability of the drug, avoids dose escalation, and enhances patient compliance (Dolati et al., 2016; Xiao et al., 2019). In this review, the manipulation strategies and anti-RA effects of nanomaterials on macrophages for the treatment of RA are analyzed, and the prospect of clinical application is also discussed.

## Strategies for the Manipulation of Macrophages by Nanomaterials

The routes of nanomaterial administration for the manipulation of macrophages in the treatment of RA mainly include intravenous injection, oral administration, percutaneous absorption, intraperitoneal injection, and intra-articular injection. In terms of the control strategy, the intravenous injection is primarily an active strategy, whereas the oral and percutaneous administrations are mainly passive strategies. Regarding the types of nanomaterials, inorganic materials are typically administered through intra-articular injection, while organic materials are mainly delivered through other routes. Based on the pathological role of macrophages in RA, the use of nanomaterials for the manipulation of macrophages mainly focuses on the early diagnosis and therapy of RA (Table 1).

**TABLE 1:** Strategies using nanomaterials to manipulate macrophages for Rheumatoid arthritis treatment.

Route of administration	Drugs/agents	Carrier systems		Consequent			Reference
				Inflammatory cytokines		Polarization and apoptosis	
		Organic material	Inorganic material				
				Reduction	Increase		
Intravenous injection	Methotrexate	FA-PPLNPs^a^	—	TNF-α, IL-6	—	—	Zhao et al. (2017)
		&HSA^a^	—	TNF-α, IL-1β, IL-6	—	Polarization	Liu et al. (2019)
		Sta-R8-FA-PLPNs^a^	—	TNF-α, IL-1β, IL-6	—	—	Zhao et al. (2018)
		DS-5β-cholanic acid^a^	—	TNF-α, IL-1β, IL-6	—	—	Heo et al. (2017)
		DS-micelle^a^	—	TNF-α, IL-1β, IL-6	—	—	Yang et al. (2017)
		FGCN^a^	—	TNF-α, IL-1β, IL-6, IL-17	IL-10	Apoptosis	Kumar et al. (2020)
		—	Fe3 + @HA MOFs^a^	TNF-α, IL-1β, IL-6	—	—	Guo et al. (2021)
		—	Au-DEN-NPs^a^	TNF-α, IL-1β, IL-6	—	—	Pandey et al. (2019)
	Methotrexate and minocycline	PLGA	—	TNF-α, IL-1, IL-6	—	—	Janakiraman et al. (2019)
	Prednisolone	HA-SLNs^a^	—	TNF-α, IL-1β, IL-6	—	—	Zhou et al. (2018)
	Prednisolone and curcumin (Chinese medicine monomer, derived from *Curcuma longa* L.)	&HAS^a^	—	TNF-α, IL-1β, IL-6	IL-10	—	Yan et al. (2019)
	Celastrol (Chinese medicine monomer, derived from *Tripterygium wilfordii* Hook. f.)	&HSA-HS15^a^	—	TNF-α, IL-1β	—	—	Gong et al. (2019)
		PEG-b-PPS	—	TNF-α, IL-1β, IL-6	TGF-β1, M-CSF	—	An et al. (2020)
	Triptolide (Chinese medicine monomer, derived from *Tripterygium wilfordii* Hook. f.)	GDR^a^	—	TNF-α, IL-1β, IL-6, IFN-γ, IL-17A	—	—	Li et al. (2020)
		&PAT	—	TNF-α, IL-1β, IL-6	—	—	Zhang et al. (2018)
	Benzoylaconitine (Chinese medicine monomer, derived from *Aconitum carmichaelii* Debx.)	mPEG-PLGA	—	TNF-α, IL-1β	—	—	Gai et al. (2020)
	Dexamethasone	HA-PNPs^a^	—	TNF-α, IL-1β	—	—	Yu et al. (2019)
	Dexamethasone palmitate	DEPE-PEG2000	—	TNF-α, MCP-1	—	—	Lorscheider et al. (2019)
	Tacrolimus	MNP^a^	—	TNF-α, IL-1β, IL-6	—	—	Li et al. (2019)
	Superoxide dismutase	F-CNM^a^	—	IL-6	—	—	Srivastava et al. (2018)
	Ag+	LA-PEG-FA^a^	—	TNF-α, IL-1β, IL-6	—	Polarization	Yang et al. (2021)
	Fumagillin prodrug	Rv-β3-FFC^a^	—	TNF-α, IL-1β, IL-6, MCP-1	—	—	Zhou et al. (2014)
	Mcl-1 siRNA	FA-PPNPs^a^	—	TNF-α, IL-1β, IL-6	—	—	Sun et al. (2019)
	IL-1β siRNA	FS14-NPs	—	TNF-α, IL-1β, IL-6	—	—	Song et al. (2019)
	NF-κB p65 siRNA	FA-PEG-liposome^a^	—	TNF-α, IL-1β	—	Polarization	Duan and Li, (2018)
	Notch1 siRNA	tGC	—	TNF-α, IFN-γ, MCP-1, IL-6, IL-12, IL-17	—	—	Kim et al. (2015)
	BTK siRNA	CLAN	—	TNF-α, IL-1β, IFN-γ	—	—	Zhao et al. (2019)
	—	—	Au-NP		—	Apoptosis	James et al. (2015)
Percutaneous absorption	Methotrexate	PLC-PEG-PLC	—	TNF-α, IL-1β, IL-6	—	—	Qindeel et al. (2020a)
		NLCs	—	TNF-α, IL-1β, IL-6	—	Apoptosis	Garg et al. (2016)
	Quercetin (Chinese medicine monomer, derived from *Quercus iberica*)	NE	—	TNF-α, IL-1β, IL-6	—	—	Gokhale et al. (2019)
Intra-articular injection	Methotrexate	—	MFC-MSNs	TNF-α, IL-1β	—	Polarization	Kim et al. (2019)
	Methotrexate and teriflunomide	—	HAP-NPs	TNF-α, IL-1β, IL-6	—	—	Pandey et al. (2021)
	Dexamethasone	—	ND-ODA	TNF-α, IL-1β	—	Polarization	Pentecost et al. (2017)
	Resveratrol (Chinese medicine monomer, derived from *Vitis* spp.)	—	QRu-PLGA-DS	TNF-α, IL-1β, IL-6	IL-4, IL-10, TGF-β	Polarization	Chen et al. (2019a)
	Clodronate	Chitosan	—	IL-8, IL-1β	—	—	Russo et al. (2016)
	TNF-siRNA	LPNs	—	TNF-α	—	—	Jansen et al. (2019)
	NOCCL	Acrylamide	—	TNF-α, IL-6	—	—	Park et al. (2017), Yeo et al. (2019)
Intraperitoneal injection	IL-10 plasmid DNA	Tuftsin-alginate NPs	—	TNF-α, IL-1β, IL-6	—	Polarization	Jain et al. (2015)
	—	—	Au25Sv5	TNF-α, IL-1, IL-6	—	—	Yuan et al. (2019a)
		—	Au29GSH7	TNF-α, IL-1β, IL-6	—	—	Gao et al. (2019)
Oral administration	Chloroquine	SLN	—	TNF-α	—	—	Bhalekar et al. (2016)
Diagnostic nanomaterials	—	&MFNPs	—	—	—	—	Periyathambi et al. (2017)

&Endogenous biomimetic materials.

a Active targeting.

FA-PPLNPs, folic acid–polyethylene glycol–poly (lactic-co-glycolic acid)–poly (cyclohexane-1,4-diylacetone dimethylene ketal)–lipid nanoparticles.

HAS: human serum albumin nanoparticles.

Sta-R8-FA-PLPNs, stearic acid-octa-arginine and folic acid decorated poly (lactic-co-glycolic acid)-PK3–based lipid polymeric hybrid nanoparticles.

DS-5β-cholanic acid, dextran sulfate-5β-cholanic acid nanoparticles.

DS-micelle, dextran sulfate-graft-methotrexate conjugate.

FGCN, folate-conjugated pH-responsive glycol-chitosan nanoparticles.

Fe3 + @HA MOFs, Fe3+ metal-organic frameworks with surface hyaluronic acid modification.

Au-DEN-NPs, nanogold core dendrimer nanoparticles.

PLGA, poly (lactic-co-glycolic acid).

HA-SLNs, solid lipid nanoparticles coated with hyaluronic acid.

PEG-b-PPS, poly (ethylene glycol)-block-poly (propylene sulphide).

GDR, pH-sensitive galactosyl-dextran-retinal.

PAT, poly-γ-glutamic acid-grafted di-tert-butyl L-aspartate hydrochloride.

mPEG-PLGA, methoxy-poly (ethylene glycol)-poly (lactide-co-glycolide).

HA-PNPs, hyaluronic acid–coated acid-sensitive polymeric nanoparticles.

DEPE-PEG2000, 1,2-distearoyl-sn-glycero-3-phosphoethanolamine-N-[methoxy (polyethylene glycol)-2000].

MNP, macrophage-derived microvesicle-coated nanoparticle.

F-CNM, folic acid-cellobiose-coated nanomatrix.

LA-PEG-FA, α-lipoyl-ω-folic poly (ethylene glycol).

Rv-β3-FFC, Rvβ3-integrin–targeted perfluorocarbon nanocarriers.

FA-PPNPs, folate acid-polyethylene glycol-poly (lactide-co-glycolide acid)-PK3 nanoparticles.

FS14-NPs, polymer–lipidoid hybrid nanoparticles.

FA-PEG-liposome, folic acid-poly (ethylene glycol)-liposome.

tGC, thiolated glycol chitosan polymers.

CLAN, cationic lipid–assisted poly (ethylene glycol)-block-poly (lactic-co-glycolic acid) nanoparticle.

PLC-PEG-PLC, polycaprolactone-polyethylene glycol-polycaprolactone triblock copolymer.

NLCs, nanostructured lipid carriers.

NE, nano-emulsion.

MFC-MSNs, manganese ferrite and ceria nanoparticle–anchored mesoporous silica nanoparticles.

HAP-NPs, hyaluronic acid coated hydroxyapatite nanoparticles.

ND-ODA, octadecylamine-functionalized nanodiamond.

QRu-PLGA-DS, quadrilateral ruthenium-poly (lactic-co-glycolic acid)-dextran sulfate nanocomposite.

LPNs, lipid–polymer hybrid nanoparticles.

MFNPs, magnetic fibrin nanoparticles.

### Manipulation of Macrophages by Intravenous Injection of Nanomaterials

Intravenous injection is the most commonly used method of administration in the clinic. It offers some advantages compared with other administration routes, such as rapid onset of effect, no first-pass effect, and absence of effects on the digestive system (Anselmo and Mitragotri, 2016). Thus far, the pathogenesis of RA remains unclear, and long-term clinical treatment is required for this disease. The pharmacological treatment of RA often involves oral administration of drugs, such as GCs, NSAIDs, and disease-modifying drugs (chemical therapy, TCM, natural cures, etc.). Nevertheless, this approach is limited by the difficulty of reaching the diseased joints and the occurrence of systemic side effects (Abbasi et al., 2019). According to statistics, 37.8% of patients discontinued treatment due to serious adverse effects. Therefore, the development of novel drug delivery systems to transport therapeutic drugs that can specifically target the synovial macrophages has become a research hot spot in the field of RA (Littlejohn and Monrad, 2018).

Nanodrug delivery systems are characterized by small particles, a large specific surface area, and a strong adsorption property. These features extend the half-life of drugs *in vivo*, thereby prolonging the duration of action and minimizing the frequency of administration (Dolati et al., 2016). Simultaneously, after being modified by chitosan, poly (ethylene glycol) (PEG), and d-α-tocopheryl polyethylene glycol 1,000 succinate or prepared from endogenous biomimetic materials, nanodrug delivery systems can selectively target inflammatory tissues by passive or active targeting, releasing drugs through a response to endogenous (pH, redox, enzymes, etc.) or exogenous (temperature, light, electric field, magnetic field, etc.) stimuli. This selective targeting improves the therapeutic effects, lowers toxicity, and reduces the incidence of side effects (Liu et al., 2020).

#### Passive Targeting Strategy

**FIGURE 2:**
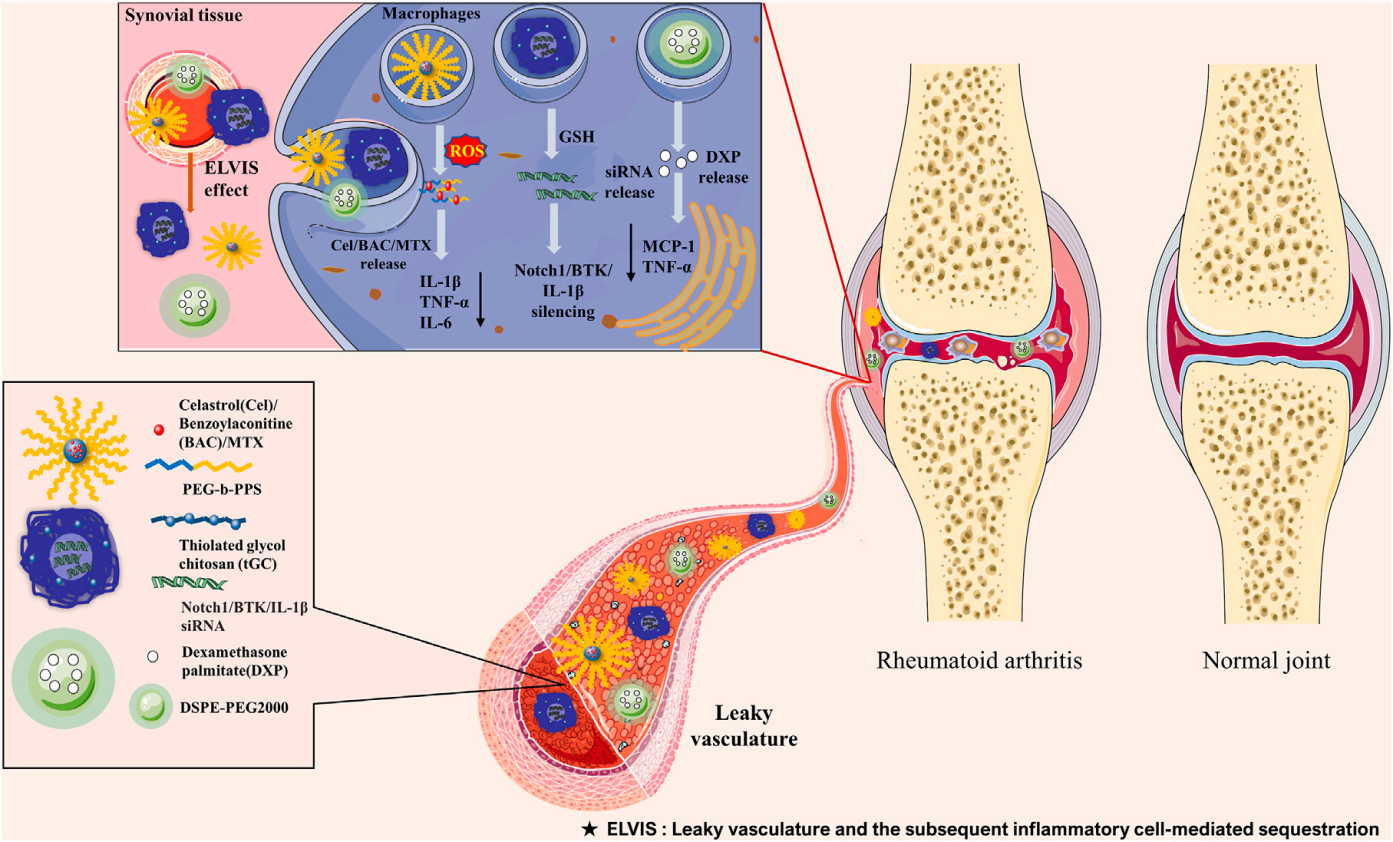
Schematic illustration of the passive targeting delivery system for the management of rheumatoid arthritis by manipulating macrophages with nanocarriers encapsulating various therapeutic agents. Polymer nanoparticles, chitosan nanoparticles, and polymeric micelles have been used for the treatment of RA. Upon intravenous administration, nanoparticles accumulate in the inflamed joints *via* the ELVIS effect. Subsequently, these nanoparticles are taken up by activated macrophages and selectively deliver Cel, BAC, MTX, DXP, and Notch1/BTK/IL-1β siRNA through pH-responsive, redox-responsive, and ROS-responsive approaches. This process reduces the release of MCP-1, TNF-α, IL-1β, and IL-6, thus alleviating the progression of RA. BAC, benzoylaconitine; BTK, Bruton’s tyrosine kinase; Cel, celastrol; DXP, dexamethasone palmitate; ELVIS, Extravasation through Leaky Vasculature and the subsequent Inflammatory cell–mediated Sequestration; GSH, glutathione; IL-1β, interleukin-1β; IL-6, interleukin-6; MCP-1, monocyte chemoattractant protein-1; MTX, methotrexate; RA, rheumatoid arthritis; ROS, reactive oxygen species; siRNA, small interfering RNA; TNF-α, tumor necrosis factor α.

The increase in vascular permeability and macrophage infiltration are the main pathological characteristics of RA, which can provide favorable conditions and target cells for nanodrug delivery systems. Persistent inflammation increases vascular permeability, which allows the nanodrug carrier to selectively accumulate and release drugs in the synovial tissue through the “ELVIS” (Extravasation through Leaky Vasculature and the subsequent Inflammatory cell–mediated Sequestration) effect, which is similar to the enhanced permeability and retention effect observed in the treatment of tumors (Kumari et al., 2016). Particle size is the key factor affecting the passive targeting strategy. This is because nanodrug carriers with a size of >200 nm and <10 nm can be eliminated by the spleen and the kidney, respectively. Thus, only nanodrug carriers with a size in the range of 100–200 nm could avoid uptake by the mononuclear phagocyte system and the reticuloendothelial system (RES), thus remaining longer in circulation in patients with RA (Kang et al., 2020) (Figure 2).

##### Inorganic Nanomaterials

Inorganic nanomaterials, such as an exogenous substance, are required to penetrate the various biological barriers *in vivo* prior to reaching the inflammatory synovial tissue of the diseased joint. Typically, after intravenous injection, nanomedicines can be easily cleared from the blood circulation through both the RES and the mononuclear phagocyte system (Yoo et al., 2010). Numerous researchers have reported that modification using hydrophilic materials can improve the surface hydrophilicity and steric hindrance of inorganic nanomaterials. These effects help to “stealth” the nanomedicines and prolong their time in circulation, providing the possibility of enrichment at sites of inflammation *via* the “ELVIS” effect (Hu et al., 2018).

###### PEG Modification

PEG is a solid hydrophilic polymer. It has been demonstrated that PEGylated nanomedicines accumulate more on inflamed synovium and are less eliminated in the spleen and the liver. In a recent study conducted by Heo et al., dexamethasone palmitate (DXP), a lipophilic prodrug of dexamethasone (Dex), and DSPE-PEG2000 were used to prepare PEGylated DXP-NPs. The preparation of DXP-NPs was based on the hydrophobic interaction between the stearic acid chain of polyethylene glycol lipids and the palmitic acid chain of DXP. It was observed that the nanomedicine was highly effective because it prevented the crystallization of DXP and avoided low drug loading and destabilization of the suspension over time. Furthermore, the PEG on the surface of NPs can make them “invisible” to the mononuclear phagocyte system and the RES and allow longer circulation in blood vessels. DXP-NPs are characterized by high vascular permeability of inflammatory joints, which can passively diffuse and accumulate in the lesion site. This accumulation led to the release of DXP, which inhibited the release of monocyte chemoattractant protein-1 (MCP-1) and TNF-α in macrophages (Lorscheider et al., 2019). Others synthesized diblock copolymer PEG-block-poly (propylene sulphide) *via* multistep chemical reaction. The blank micelle (B-PEPS) was prepared through self-assembly of a copolymer; subsequently, celastrol (Cel) was added to prepare Cel-loaded diblock copolymer nanomicelles (C-PEPS). The hydrophilic PEG block could increase the circulation time of nanomicelles, and the hydrophobic poly (propylene sulphide) block permits reactive oxygen species (ROS)-sensitive reactions. After reaching the inflammatory site through the “ELVIS” effect, the nanomedicine rapidly released Cel *via* the ROS-responsive approach, which prevented the cleavage and activation of nuclear factor-κB (NF-κB) and Notch1. This process suppressed M1 macrophage activation and the release of pro-inflammatory cytokines (e.g., TNF-α, IL-6, and IL-1β), whereas it enhanced the release of anti-inflammatory cytokines [e.g., TGF-β1 and macrophage colony-stimulating factor (M-CSF)] (An et al., 2020). Another monomer of TCM, benzoylaconitine (BAC), was also prepared in PEG-modified nanomedicine for the manipulation of macrophages in the treatment of RA. Researchers designed the methoxy-PEG-poly (lactide-co-glycolide) (mPEG-PLGA) copolymer through ring-opening polymerization and dissolved it in dimethyl sulfoxide and BAC in methanol. Finally, the mPEG-PLGA and BAC solutions were mixed to prepare BAC-loaded mPEG-PLGA NPs (NP/BAC). Through the “ELVIS” effect, the nanomedicine aggregated in the inflammatory joints and released BAC to reduce the overexpression of NF-κB p65, block the production of pro-inflammatory cytokines, and alleviate the development of inflammation (Gai et al., 2020). Small interfering RNA (siRNA)-mediated gene silencing has been used in the treatment of autoimmune diseases, such as RA. However, due to its poor stability and low permeability, chemical modification is required to overcome these limitations (Yu et al., 2020). Bruton’s tyrosine kinase (BTK) is an important macrophage kinase which can promote the polarization of pro-inflammatory macrophages. Moreover, downregulating the expression of BTK in macrophages can reduce inflammatory cytokines, inhibit the development of RA, and alleviate symptoms of arthritis (Rip et al., 2018; Zhao et al., 2019). More recent studies conducted by Zhao et al. employed mPEG5K-b-PLGA11K and cationic liposome (DOTAP) to prepare cationic lipid–assisted NPs (CLANs) using a double-emulsion solvent evaporation method. Next, siBTK was encapsulated into CLANs to prepare CLANsiBTK. The nanomedicine specifically targets the activated macrophages *via* the “ELVIS” effect to downregulate the expression of BTK, thereby reducing the release of inflammatory cytokines [e.g., TNF-α, IL-1β, and interferon-γ (IFN-γ)] (Zhao et al., 2019).

###### PEG Analogue Modification

PEGylated polymer or aqueous amphiphilic block copolymers, such as poly (ethylene oxide)-poly (propylene oxide)-poly (ethylene oxide) (PEO-PPO-PEO), can effectively prolong the blood circulation time and achieve long-term effects as the matrix of NPs. Scholars modified NPs with amphiphilic PEG analog F127 (PEO-PPO-PEO) to manipulate macrophages for delivering siIL-1β. F127 is an amphiphilic triblock copolymer composed of PEO and PPO. When the concentration reaches a specific critical value, the PEO-PPO-PEO block copolymer can self-assemble into a core–shell micelle structure due to the different hydrophilicities of PEO and PPO in water. In this structure, the PPO and PEO segments form the core and the shell of the micelle, respectively. Researchers injected a mixture of spermidine lipid (S14) and F127 into acetate buffer to synthesize polymer–lipid mixed NPs (FS14-NP) using the nanoprecipitation method. The nanomedicine selectively accumulated in the diseased joints through the “ELVIS” effect and was endocytosed through the natural phagocytosis of activated macrophages, delivering siIL-1β to suppress ankle swelling, bone erosion, and cartilage destruction (Song et al., 2019). Janakiraman et al. (2019) also developed PLGA NPs delivering MTX and minocycline. PEG analog d-α-tocopheryl polyethylene glycol 1,000 succinate was used as an absorption promoter, stabilizer, solubilizer, and emulsifier to prolong the circulation time of the drug. Surfactant Span-80 was added to prevent the aggregation of particles. As expected, the NPs were absorbed by activated macrophages, releasing MTX and minocycline in inflamed joints. Owing to its good biocompatibility and biodegradability, PLGA can enhance the release of medicine, which inhibited pro-inflammatory cytokines. In addition, minocycline controls infections caused by Gram-positive and Gram-negative bacteria and effectively treats RA associated with severe infection.

###### Chitosan Modification

As a natural polysaccharide rich in amino groups, chitosan is widely used in the biomedical field owing to its good biocompatibility, bioactivity, lack of toxicity, and biodegradability. Recently, the study of amphiphilic chitosan derivatives has attracted growing attention. Amphiphilic chitosan–based copolymers (e.g., glycol chitosan), formed by the hydrophobic modification of chitosan, can be dissolved in any aqueous solution and are characterized by prolonged circulation time (Jiménez-Gómez and Cecilia, 2020). Kim et al. (2015) have encapsulated the siRNA targeting the Notch pathway into thiolated glycol chitosan polymer, which realized the long cycle characteristics *in vivo* and assembled explicitly in RA joints. Following phagocytosis by activated macrophages, disulfide bonds in nanomedicine are broken in the presence of reducing agents such as glutathione (r-glutamyl cysteingl + glycine; GSH) in the cytoplasm. This results in the release of siRNA to silence the Notch pathway, thereby delaying bone erosion and cartilage injury.

##### Endogenous Biomimetic Nanomaterials

Recent evidence suggests that PEGylated modified polymer NPs cause a robust immune response, which can accelerate the blood clearance effect (Mohamed et al., 2019). Thus, a natural particle-based biomimetic drug delivery system was created, which disguised NPs as autologous components to reduce their immunogenicity, escape immune clearance, prolong circulation time, increase accumulation, improve therapeutic efficiency, and reduce toxicity and side effects (Jin et al., 2018). L-aspartic acid is an important amino acid with attractive properties for iron oxide NP functionalization, low cost, and high biocompatibility. It is widely used in the food, medical, and chemical industries; its application in medicine is particularly noteworthy (Salehiabar et al., 2018). In a study, triptolide (TPT), a monomer of TCM, was carried by poly-γ-glutamic acid–grafted di-tert-butyl L-aspartate hydrochloride, which was prepared *via* the amidation reaction of aspartic acid with poly-γ-glutamic acid. NPs concentrated in the diseased joints through the “ELVIS” effect were uptaken by activated macrophages and released TPT to reduce pro-inflammatory cytokines (TNF-α, IL-1β, and IL-6) (Zhang et al., 2018). Other researchers also used macrophage-derived microvesicles combined with nanocarriers to prepare macrophage-derived microvesicle-coated NPs (MNPs). NPs can be recognized as endogenous vesicles by connecting with natural cell membranes, reducing the elimination of MNPs by the host, and prolonging their circulation. They can also simulate macrophages through CD44 or macrophage antigen 1 (Mac-1) recognition, adhere to the endothelium to target the inflammatory site of RA for the transportation of drugs, and mimic a macrophage to combine with M-CSF and NF-κB ligand–receptor activator (RANKL) for the inhibition of osteoclastogenesis (Li et al., 2019).

#### Active Targeting Strategy

##### Direct Manipulation Strategy

**FIGURE 3:**
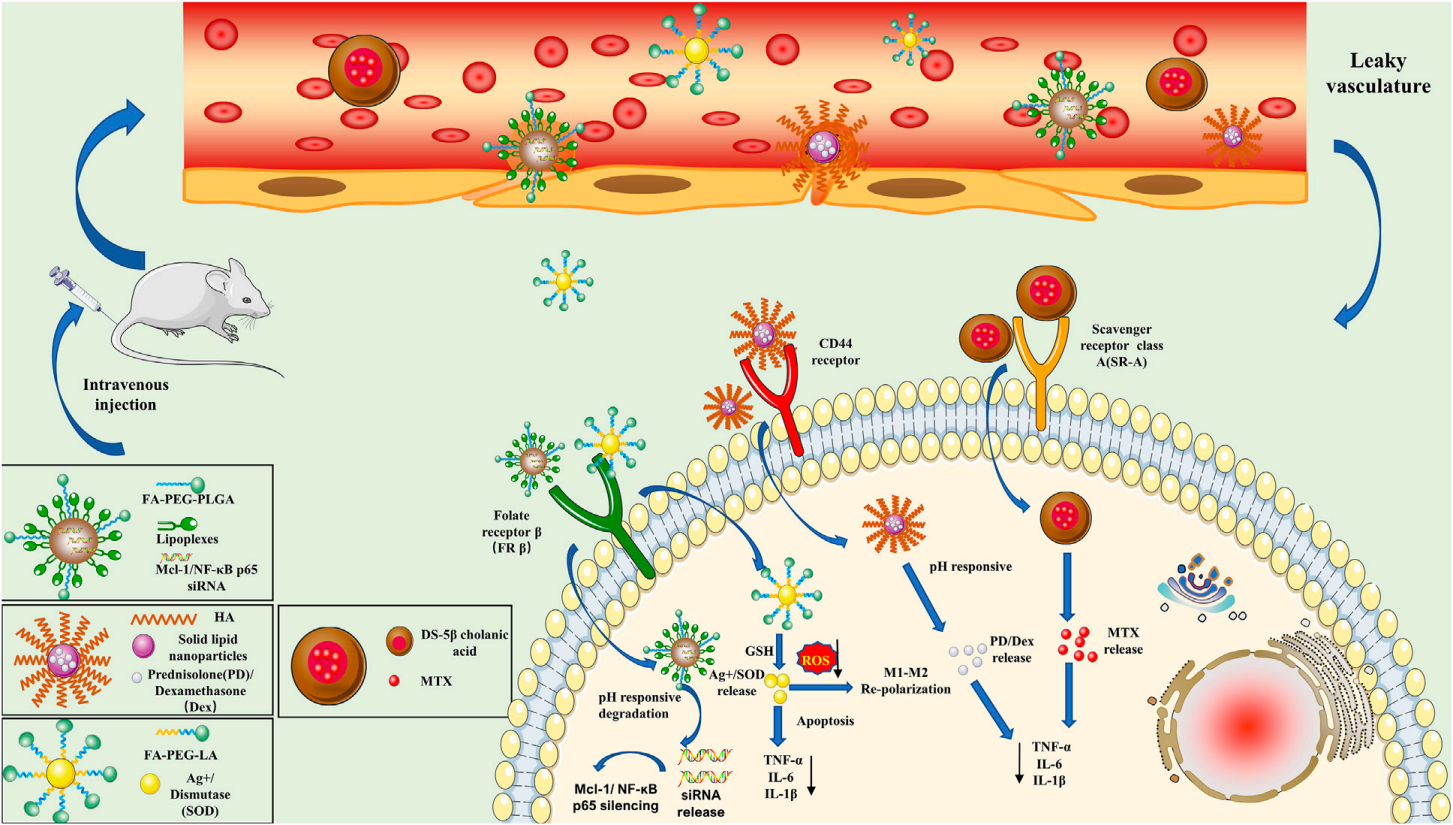
Schematic illustration of the active targeting nanoparticles (NPs) approach in rheumatoid arthritis. (1) Mcl-1/NF-κB p65 siRNA-NPs are fabricated by encapsulating poly-siRNA into lipid–polymer hybrid NPs. Following systemic administration, NPs accumulate in the inflamed joints by taking advantage of the leaky blood vessels and selectively delivering Mcl-1/NF-κB p65 siRNA into activated macrophages through folate receptor–mediated endocytosis. (2) Ag+/SOD-loaded NPs dissolve and release Ag+/SOD in response to intracellular GSH, which synergistically induces apoptosis in M1 macrophages and scavenges ROS to cause the polarization of M1 macrophages to the M2 phenotype in inflamed synovial joints. (3) PD/Dex is wrapped in HA-coated solid lipid NPs to prepare NPs for pH-responsive drug release. HA coating allows NPs to enter macrophages through CD44 receptor–mediated endocytosis, thereby reducing the release of TNF-α, IL-1β, and IL-6. (4) Dextran sulfate acts as a ligand for macrophage scavenger receptor class A, which is overexpressed by activated macrophages. Nanoparticles enter activated macrophages to release MTX and inhibit the release of TNF-α, IL-1β, and IL-6. Dex, dexamethasone; FA-PEG-PLGA, folic acid-poly (ethylene glycol)-poly (lactide-co-glycolide); FA-PEG-LA, FA-PEG-lipoic acid; GSH, glutathione; HA, hyaluronan; IL-1β, interleukin-1β; IL-6, interleukin-6; Mcl-1, myeloid cell leukemia-1; MTX, methotrexate; NF-κB, nuclear factor-κB; PD, prednisolone; ROS, reactive oxygen species; siRNA, small interfering RNA; SOD, superoxide dismutase; TNF-α, tumor necrosis factor α.

Although the nanodrug delivery system can passively deliver drugs to RA lesion areas through the “ELVIS” effect, it cannot be completely ingested by macrophages. It can also bind with other nontarget cells, which has a limited effect on the manipulation of macrophages in RA (Gaspar et al., 2019). Active targeting strategies were developed to overcome shortcomings and enhance the aggregation of nanomedicines at the target site. The primary process of active targeting is achieved *via* surface modifications of nanomedicines with target-specific ligands (Shi and Lammers, 2019). Researchers used small-molecule ligands (i.e., folate, class A scavenger, and galactose) for the modification of nanodrugs to target a variety of specific receptors that are overexpressed on the surface of macrophages, such as folate receptors (FRs), class A scavenger receptors (SR-As), and galactose receptors (Pirmardvand Chegini et al., 2018); they constructed a nanodrug delivery system *via* active targeting of macrophages (Figure 3).

###### Receptor Targeting Strategy

Macrophages are a type of special immune cell with phagocytic function. Studies have shown that various membrane receptors can mediate the endocytosis of specific ligands, such as FRs and SR-As. It was observed that the FR, SR, CD44 receptor, and galactose receptor are overexpressed on the surface of macrophages, mediating the endocytosis of folate, acetylated low-density lipoprotein, hyaluronan (HA), and galactose. The nanodrug carriers can manipulate macrophages through conjugation with cell surface–specific ligands, thus exerting their therapeutic effects (Pirmardvand Chegini et al., 2018; Tardito et al., 2019).

###### FR

The FR is a receptor on the cell surface that can be used as a target site for the treatment of RA. There are four common subtypes of FRs, namely, FR-α, FR-β, FR-γ, and FR-δ. FR-α is used for the targeted therapy of tumors. FR-β is overexpressed by activated macrophages in inflammatory synovial tissues and has been used for the targeted diagnosis and treatment of RA (Kumar et al., 2019). Duan et al. designed the PEGylated liposome targeting macrophages; siRNA/calcium phosphate NPs were loaded in its core and the antirheumatic drug MTX was loaded in the lipid shell. The nanomedicine possessed better transmembrane transport capacity and delivery characteristics than other siRNA nanocarriers (e.g., polyethyleneimine, dendrimers, and chitosan). Moreover, the PEGylated liposome combined with folic acid (FA) could specifically target activated macrophages *via* FRβ. This resulted in silencing the expression of NF-κB p65 (a classical inflammatory signaling pathway) to downregulate inflammatory cytokines and promote the polarization of M1 macrophages to M2 for the treatment of RA (Duan and Li, 2018). Zhao et al. (2017) also used FA as a ligand to deliver nanomedicines to activated macrophages in inflammatory joints. The difference is that the investigators used the acid-sensitive carrier polyketide poly (cyclohexane-1,4 diyl acetone dimethylene ketal) (PCADK) for acid-responsive targeted drug delivery. As a pH-responsive material, PCADK is stable in a neutral environment, prolonging its circulation time in plasma. In contrast, it rapidly degrades in the acidic environment to release drugs. However, PCADK also has shortcomings. Owing to the strong hydrophobicity, the hydrolysis half-life of the nanocarrier is longer, complicating its removal from the body (Wang et al., 2015; Zhao et al., 2017). Later, researchers synthesized a novel polyketal (PK3) by adding diols with strong hydrophilicity during the reaction, which overcame the limitations of PCADK (Guo et al., 2016). Zhao et al. added PK3, FA-PEG-PLGA, egg phosphatidylcholine, stearic acid-octa-arginine (Sta-R8), and MTX to the mixed solvent of dichloromethane and acetone to prepare hybrid pH-responsive lipid polymer NPs (Sta-R8-FA-PLPNs/MTX). PEG-modified NPs selectively accumulate in inflammatory joints, penetrate the cell membrane through the effects of Sta-R8, and are subsequently phagocytosed by activated macrophages through FR-mediated endocytosis. PK3 can also act as a pH-sensitive switch that degrades under acidic conditions, thereby releasing MTX to inhibit pro-inflammatory cytokines (Zhao et al., 2018). The myeloid cell leukemia-1 (Mcl-1) protein, a member of the antiapoptotic B-cell lymphoma-2 (Bcl-2) family of proteins, was overexpressed in synovial macrophages of patients with RA. Mcl-1 protects macrophages from apoptosis by blocking the activation of the proapoptotic molecule Bax. NPs can induce apoptosis by silencing the expression of the Mcl-1 protein in activated macrophages to exert therapeutic effects in RA (Xiang et al., 2018). Therefore, Sun et al. (2019) also used the same strategy as Zhao et al. (2018) for the delivery of Mcl-1/siRNA to induce apoptosis and exert its anti-RA effects. Moreover, scholars incorporated MTX (an antirheumatic drug) into FA-conjugated glycol chitosan NPs to treat RA. The NPs could induce the mitochondrial membrane potential, increase the levels of nitric oxide (NO), reduce the antioxidant status, and induce apoptosis in macrophages (Kumar et al., 2020). In addition to the acid-sensitive drug release of NPs in macrophages, some scholars also used GSH for redox-responsive drug release. Researchers used FA-modified silver NPs to actively target M1 macrophages, leading to the release of Ag^+^ under the action of intracellular GSH. This caused apoptosis in M1 macrophages and ROS clearance so as to allow M1-to-M2 repolarization, thereby alleviating inflammation to achieve higher efficacy and biosafety (Yang et al., 2021). This study is the first to use bioactive nanomaterials (without drug loading) for the manipulation of macrophages in the treatment of RA, providing a new idea for treating RA with nanomaterials. Superoxide dismutase (SOD) is a metal enzyme widely distributed in animals, plants, and microorganisms which can catalyze the disproportionation reaction of superoxide radicals in organisms. It is a natural scavenger of O_2_ in the body that plays a significant role in the immune system, which is used to treat RA and other diseases (Wang et al., 2018). Srivastava et al. (2018) added propylene sulfide to Pluronic F-127 using the emulsion ring-opening polymerization method to prepare a propylene sulfide nano-matrix. Subsequently, they used the propylene sulfide nano-matrix and fiber disaccharide solution as raw materials to prepare a cellobiose-coated nano-matrix (CNM). Targeting ligand FA was efficiently conjugated to SOD using the linking agent 1-ethyl-3-(3-dimethylaminopropyl) carbodiimide to prepare F-SOD, the CNM was dispersed in the F-SOD solution to adsorb SOD, and the cellobiose-coated FA-SOD NPs (FECNM) were prepared. The porous coating of cellobiose in the NPs exerts a strong adsorption effect on the enzyme and could effectively adsorb SOD. Simultaneously, the addition of propylene sulfide can improve the oxidation resistance of NPs; the oxidant is converted into peroxide, and the hydrophobicity is transformed into hydrophilicity. FECNM selectively accumulates in inflammatory joints through the “ELVIS” effect and targets activated macrophages *via* FRβ, delivering SOD to improve the antioxidant response of macrophages and reduce pro-inflammatory cytokines. The NPs can also be used as an enzyme library of acid-unstable enzymes in the controlled form for the efficient treatment of RA.

###### CD44 Receptor

CD44 is a type of adhesion receptor widely distributed in epithelial cells, activated lymphocytes, and tumor cells. It was found that the expression of CD44 in the inflammatory environment is increased in macrophages, fibroblasts, and lining cells. HA is a natural polysaccharide widely used in drug delivery systems or tissue engineering as the ligand for the CD44 receptor (Jiao et al., 2016; Pandey et al., 2017). Researchers have prepared HA-solid lipid NPs/prednisolone (HA-SLNs/PD) by wrapping GC PD in SLNs coated with HA. SLNs possess high biocompatibility, physical stability, and drug loading and the ability to protect unstable drugs from degradation. The inclusion of PD in SLNs can enhance the accumulation of drugs in arthritic joints, prolong circulation time in the blood, and reduce severe adverse effects. HA-modified NPs can enter the target cells through CD44 receptor–mediated endocytosis, thereby reducing joint swelling, bone erosion, and serum inflammatory cytokines in experimental animals with RA (Zhou et al., 2018). Other researchers also developed HA-modified NPs for delivering Dex. However, in terms of drug release, researchers mainly used the pH-sensitive polyketone PCADK as the acid-sensitive carrier to release Dex, thereby reducing the levels of inflammatory cytokines (Yu et al., 2019).

###### SR

SR is a macrophage surface glycoprotein that can effectively mediate the uptake of oxidized and acetylated low-density lipoproteins. At present, SR has been widely used in the treatment of atherosclerosis. Studies have revealed that it also plays a crucial role in RA. Researchers conjugated hydrophobic 5β-cholanic acid to a hydrophilic dextran sulfate (DS) skeleton to synthesize the amphiphilic DS derivative, which could self-assemble in the aqueous status. Next, MTX was loaded into DS NPs to prepare MTX-loaded DS NPs by dialysis. As the ligand for the SR-A receptor, DS has been utilized as a drug cargo to bind to the SR-A receptor on the activated macrophages. The study demonstrated that the MTX-loaded DS NPs could accumulate in the inflammatory site through SR-A–mediated endocytosis, releasing MTX to inhibit TNF-α, IL-1β, and IL-6 in RA (Heo et al., 2017). Other scholars have synthesized the macrophage-targeted DS–MTX conjugate (DS-*graft*-MTX) and the untargeted dextran−MTX prodrug (De-*graft*-MTX) to determine differences in the treatment of RA. Notably, the diameters of these two kinds of MTX micelles are < 100 nm to avoid the rapid elimination by the RES and to selectively concentrate on the inflammatory joints of RA through the “ELVIS” effect. As expected, the degree of accumulation and the anti-arthritic effect of DS-*graft*-MTX in RA were markedly higher than those of De-*graft*-MTX (Yang et al., 2017). Based on these findings, active targeting of macrophages by receptors has substantial clinical advantages and application prospects in the treatment of RA.

###### Galactose Receptor

The galactose receptor is a C2 type lectin, which is also overexpressed on macrophages, monocytes, dendritic cells, hepatocytes, and other cells. It can mediate the binding, phagocytosis, and clearance of microorganisms by macrophages. Studies have found that this receptor can specifically recognize galactose, glucose, and their conjugates to mediate cellular phagocytosis and remove foreign bodies *in vivo*. Li et al. (2020) formed pH-sensitive galactose-based dextran-retinal (GDR) NPs, using a dual-loading strategy to combine all-trans-retinal (all-trans retinoic acid prodrug) with a dextran backbone through a pH-sensitive hydrazone bond; the NPs were modified with galactose. After self-assembly, TPT was encapsulated into the hydrophobic core by dialysis to prepare the dual drug–loaded NPs (galactose-based dextran-retinal-TPT). They determined that the NPs accumulated in the RA joints through the “ELVIS” effect and were subsequently uptaken by galactose receptor–mediated endocytosis to release TPT and all-trans-retinal, in turn suppressing the production of pro-inflammatory cytokines in macrophages. Interestingly, all-trans-retinal is oxidized to all-trans retinoic acid, which can reduce the infiltration of macrophages and inhibit the differentiation of osteoclasts; both play a synergistic role in the treatment of RA.

###### Non-Receptor Targeting Strategy

In addition to the active manipulation of macrophages *via* linking with macrophage receptors through ligands, studies have reported some non-receptor manipulation strategies for drug-loaded NPs. For example, albumin is the most abundant protein in the blood and is characterized by good stability, biocompatibility, and biodegradability and a high drug encapsulation rate. It was used as a drug delivery carrier material in the 1990s, widely employed in the treatment of cancer, RA, diabetes, and other diseases. Human albumin NPs are non-immunogenic, thereby avoiding recognition by the RES and prolonging their circulation time in the bloodstream to exert their therapeutic effects in RA joints (Tan and Ho, 2018; Liu et al., 2019; Lamichhane and Lee, 2020). Gong et al. (2019) dissolved Cel and soybean oil in methylene chloride to provide an organic phase and added HS15 to 20% human serum albumin (HSA) dispersed in distilled water to achieve an aqueous phase. Cel-HSA-HS15 NPs are prepared by adding the aqueous phase to the organic phase. Because of the “ELVIS” effect and the targeting ability of albumin, NPs can accumulate in the site of inflammation and are subsequently phagocytosed by the activated macrophages *via* macropinocytosis and clathrin-mediated endocytic pathways. The anti-arthritis drug Cel is released, manipulating them to inhibit pro-inflammatory cytokines (e.g., TNF-α and IL-1β). Of note, the drug-loaded NPs can also improve RA-related lung diseases. Yan et al. (2019) also used albumin as a nanomaterial for the delivery of PD and the monomer curcumin (CU) *via* a dual drug delivery strategy to manipulate macrophages in the treatment of RA. First, PD and CU were dissolved in acetone. Oleic acid (OA) was added to prepare PD-OA and CU-OA. PD-OA, CU-OA, purified yolk lecithin (E80), and cholesterol were dissolved in chloroform. Finally, HSA was added to prepare dual drug–loaded albumin NPs (N-PD/CU) by forming new disulfide bonds in the free sulfhydryl groups of albumin using the high-pressure homogenization method. These drug-loaded NPs can compensate for the poor bioavailability of PD and CU. The disulfide bond of NPs can rapidly release PD and CU in the presence of GSH. Interestingly, PD in NPs can reduce pro-inflammatory cytokines, while CU can increase the anti-inflammatory cytokine IL-10. Both play a synergistic anti-RA role by regulating the balance of pro- and anti-inflammatory cytokines. In the two studies above regarding albumin drug-loaded NPs, the researchers proposed that albumin NPs can accumulate in RA lesion sites through the “ELVIS” effect due to their non-immunogenic characteristics and can subsequently be phagocytosed by the activated macrophages, resulting in the manipulation of these cells. This strategy appears to be a passive targeting method. Nevertheless, Liu et al. investigated the drug delivery system based on albumin and suggested that the use of these drug-loaded NPs was an active targeting strategy. Researchers have confirmed that albumin nanocarriers can accumulate in RA synovial tissue due to the secreted protein acidic and rich in cysteine (SPARC). SPARC is a member of the extracellular matrix that is overexpressed in the synovial membrane of patients with RA and mice with collagen-induced arthritis. It has a strong intrinsic affinity for albumin, which can improve the enrichment of albumin carrier drugs in the RA synovial membrane and realize the active targeting of drug-loaded albumin NPs (Nakazawa et al., 2020). The study also proved that HSA, as a target, binds to SPARC; this leads to the accumulation of drug-loaded human albumin NPs in the arthritis synovium and the release of drugs under acidic conditions (Liu et al., 2019).

##### Indirect Manipulation Strategy

Indirect manipulation strategies have also been used in the treatment of RA. The NPs prepared by Zhou et al. do not directly manipulate macrophages by delivering drugs. Instead, they indirectly suppress inflammation through their transfer to angiogenic vessels to generate endothelial NO through a local nitrosative response. In their test, the researchers saponified fumagillin dicyclohexylamine salt to fumagillol. They subsequently esterified the product with 1-palmitoyl-2-azelaoyl-sn-glycero-3-phosphocholine to prepare the Sn-2 phospholipase labile fumagillin prodrug (Fum-PD). Finally, the Fum-PD was wrapped in Rvβ3 integrin–targeted perfluorocarbon NPs to prepare Rvβ3-targeted fumagillin prodrug–loaded perfluorocarbon (Fum-PD-FFC) NPs. Following its entry into endothelial cells, Fum-PD-FFC was cut by phospholipase at the Sn-2 site to release the active drug. Fum-PD induced the release of NO, which in turn activates AMP-activated protein kinase (AMPK), inhibits mammalian target of rapamycin (mTOR), enhances autophagy flux, and ultimately suppresses the NF-κB signaling pathway and the release of inflammatory cytokines (Zhou et al., 2014).

##### Inorganic Nanomaterials

In recent years, the development of inorganic nanomaterials has been rapid. These materials offer the advantages of easy structure adjustment and modification, superior chemical stability, good biological safety, and high drug-loading rate compared with the organic polymer nanomaterials widely used in targeted drug delivery, imaging diagnosis, and collaborative drug therapy. Researchers engineered gold (Au) nanomaterials to manipulate macrophages for the treatment of RA through intravenous administration. The results showed that Au-NPs could enter macrophages *via* a receptor-mediated, clathrin-dependent endocytosis pathway and inhibit thioredoxin reductase (TrxR), which is involved in the redox activity of macrophages. Inhibition of TrxR can induce oxidative stress and promote apoptosis in macrophages. The uptake of TrxR is greater than that of auranofin, which is transported into cells using the sulfhydryl shuttle model. Simultaneously, the surface of Au-NPs can be modified with a Au–sulfur (Au–S) bond *via* chemical grafting or electrostatic coating, which endows nanomaterials with multiple biological functions (James et al., 2015). In the study conducted by Pandey et al., the Au-NP was modified with a thiolated dendritic polymer to produce nanogold core dendrimer NPs (Au-DEN-NPs). The hydroxyl groups on the surface of the NPs were conjugated with MTX, and near-infrared active bioactive IR780 was encapsulated to offer a photothermal benefit. MTX is an FA analog with similar physical, chemical, and structural properties to those of FA. Therefore, researchers used MTX instead of FA as the target ligand to achieve selective localization through upregulated FA receptors in arthritis tissue. The near-infrared irradiation increased the temperature of the exposed environment and mediated the release of MTX. Simultaneously, the combination of MTX and ROS produced by IR-780 near-infrared laser irradiation exerts a synergistic effect to inhibit pro-inflammatory cytokines (Pandey et al., 2019). Metal-organic frameworks (MOFs) are organic–inorganic hybrid materials with intramolecular pores assembled from organic ligands, metal ions, or clusters through coordination bonds. As new nanomaterials, MOFs are characterized by high drug-loading capacity, simple preparation, and good biodegradability and are widely used in various fields (Ibrahim et al., 2017). Guo et al. (2021) encapsulated MTX into HA-modified MOFs to prepare MTX-loaded MOFs, which markedly accumulated in arthritis joints. Researchers used tannic acid as a linker in the drug-loaded NPs for conjugation with MTX through an ester bond to improve the stability of NPs. By interfering with the expression of TNF-α, IL-1β, and IL-6 in macrophages, NPs can alleviate inflammation and bone destruction.

### Manipulation of Macrophages by Nanomaterials Administered Through Percutaneous Absorption

Percutaneous absorption represents an extremely attractive and innovative route of administration. It offers numerous advantages compared with oral and intravenous administration, namely, patient convenience, avoidance of first-pass metabolism and gastrointestinal tract irritants, maintenance of constant target drug concentration, and reductions in the frequency of administration and side effects (Qindeel et al., 2020b). However, due to the barrier function of the stratum corneum, penetration of the skin by traditional percutaneous absorption products may be difficult, thereby limiting their clinical use. Nanomedicine attracts considerable attention because of its advantages (i.e., improving chemical stability and promoting percutaneous absorption) and has thus become an ideal transdermal drug delivery method (Seah and Teo, 2018). Researchers developed polycaprolactone-PEG-polycaprolactone–based nanomicelles for the transdermal delivery of MTX. Subsequently, the nanomicelles were loaded into a carbopol 934–based hydrogel with eucalyptus oil to prepare an MTX nanomicelle–loaded hydrogel. As an enhancer of penetration, eucalyptus oil can improve the skin permeability and release of MTX. This allows MTX nanomicelles to selectively accumulate in inflammatory joints and be internalized by activated macrophages through clathrin- and scavenger receptor–dependent endocytic pathways (Qindeel et al., 2020a). In another study, researchers developed a hydrogel with co-incorporated chemical enhancers (CEs) and lipid nanocarriers for the efficient transdermal delivery of MTX (MTX-nanostructured lipid carrier gel). Surfactant Kolliphor® P188 was added to reduce the particle size and improve the encapsulation efficiency. The results confirmed that the nano-size of nanostructured lipid carriers could maintain close contact with keratinocytes. The synergistic effect of the CE and lipid mixture can enhance flux and facilitate percutaneous absorption (Garg et al., 2016). In addition, Gokhale et al. prepared quercetin (QCT)-loaded nanoemulsion (NE)-based gel (QCT-NE gel) for the treatment of RA. QCT-NE, prepared by spontaneous emulsification techniques, was dispersed in a carbomer 940 gel matrix to produce a QCT-NE gel. Next, Tween 20 and PEG-400 were added to increase the solubilization ability and the permeability coefficient of NE. Owing to its small droplet size, NE significantly increases the permeation rate since the nano-sized droplets can transfer the drug through the skin barrier and rapidly move into the stratum corneum. Furthermore, water in the gel system hydrates the skin, leading to cell expansion in the stratum corneum and broadening the drug channel, thereby improving cumulative penetration. Following the transdermal administration of NPs, QCT was released through skin-specific accumulation in the arthritis site to inhibit the release of pro-inflammatory cytokines from activated macrophages (Gokhale et al., 2019). At present, there are few studies on percutaneous absorption drug delivery pathways for the treatment of RA. Due to the skin barrier function, penetration and absorption through the skin may be challenging for most drug molecules. Mice are often selected for animal experiments in research concerning the percutaneous absorption drugs. However, because of the thin layer of the mouse skin and the differing characteristics from those of humans, animal models may not yield robust results.

### Manipulation of Macrophages by Nanomaterials Administered Through Intra-Articular Injection

**FIGURE 4:**
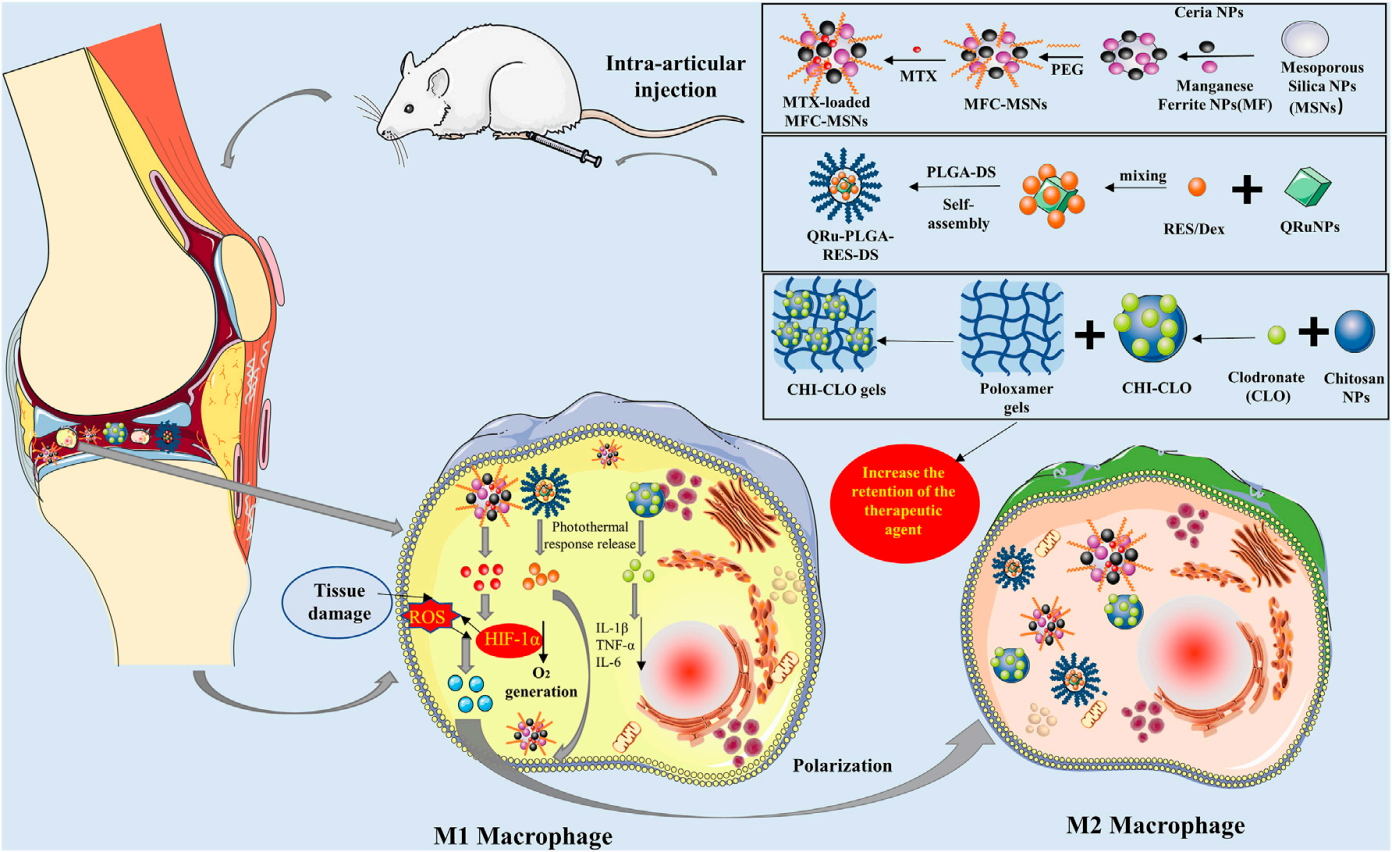
Preparation and application of MTX-loaded MFC-MSN nanoparticles (NPs), QRu-PLGA-RES-DS NPs, CHI-CLO gels, and intra-articular injection of NPs into an RA model mouse. (1) MFC-MSNs synergistically scavenge ROS and produce O_2_, leading to the polarization of pro-inflammatory M1 macrophages to the anti-inflammatory M2 phenotype in hypoxic and inflamed synovial joints. (2) As photothermal agents, the Ru NPs control the precise release of the RES through the photothermal effect and achieve high-efficiency polarization of M2 type macrophages for treating RA. (3) CHI-CLO NPs were added into the poloxamer gel matrix for intra-articular administration, which increased CLO retention in the joint, improved the therapeutic effect, reduced the side effects, and inhibited the release of IL-1β, TNF-α, and IL-6 in macrophages. CHI-CLO, chitosan-clodronate; IL-1β, interleukin-1β; IL-6, interleukin-6; MTX, methotrexate; MFC-MSNs, manganese ferrite and ceria nanoparticle–anchored mesoporous silica nanoparticles; PEG, poly (ethylene glycol); QRu-PLGA-RES-DS, quadrilateral ruthenium-poly (lactic-co-glycolic acid)-resveratrol-dextran sulfate; RA, rheumatoid arthritis; ROS, reactive oxygen species; Ru, ruthenium; TNF-α, tumor necrosis factor α.

Intra-articular injection is currently a common method used in the treatment of RA; it changed the distribution pattern by directly delivering the drug in the synovial joint. This approach avoids physiological barriers during transport, ameliorating the dosage and safety profile of drugs (Rai and Pham, 2018). However, due to the fast metabolism of the joint cavity, the injected drug is rapidly cleared, resulting in a short retention time and short-acting effect. Thus, frequent injections are required to achieve therapeutic effects, leading to local pain, swelling, and infection. Owing to its targeting properties, the nanodrug delivery system can be selectively adsorbed on inflammatory synovial tissue, overcoming this defect (Rahman et al., 2017). The carriers used for intra-articular injection are mostly inorganic materials (Figure 4). Kim et al. synthesized manganese ferrite NPs *via* thermal decomposition of manganese oleate and iron oleate complex and dissolved cerium (III) acetate and oleylamine in xylene to synthesize ceria NPs. Finally, BMPA-capped manganese ferrite NPs were synthesized by dissolving BMPA, citric acid, and capped manganese ferrite NPs in a mixture of chloroform and N, N-dimethylformamide. Similarly, BMPA-capped ceria NPs were synthesized using the same method. Large pore-sized mesoporous silica NPs and MTX were added to these two NPs to prepare manganese ferrite and ceria NP–anchored mesoporous silica NPs. The NPs were directly injected into the articular cavity to manipulate macrophages through phagocytosis. Through the synergistic effect of manganese oleate, cerium, and MTX, these NPs inhibit the expression of hypoxia-inducible factor (HIF-1α), increase ROS clearance, release O_2_, and induce the transformation of M1 macrophages to M2 macrophages (Kim et al., 2019). A multifunctional nanotherapeutic system based on another inorganic material, ruthenium (Ru), is also used to manipulate macrophages for the treatment of RA. Chen et al. (2019) formed QRu-PLGA-RES NPs by mixing the quadrilateral ruthenium (QRu) NPs, PLGA, and resveratrol; QRu and the heat-sensitive molecule PLGA were in the core and the shell of the NPs, respectively. Owing to the high affinity of DS for SR, NPs can be engulfed by activated macrophages to release resveratrol in the presence of exogenous light and induce the polarization of M1 macrophages to M2 macrophages. Recently, scholars used surface-modified nanodiamond (ND) as a nanocarrier for the delivery of Dex in the treatment of RA. In their test, researchers reacted ND-COOH with thionyl chloride in N, N-dimethylformamide to synthesize ND-COCl, which was reacted with octadecylamine (ODA) to replace the -Cl group with a -nh2 group; finally, Dex was added to prepare ND-ODA-Dex. The surface modification of ODA can significantly increase the adsorption of Dex. ND-ODA-Dex can be engulfed by activated macrophages after intra-articular injection, thereby inhibiting the release of pro-inflammatory cytokines and promoting the polarization of M1 macrophages to M2 macrophages. Researchers also found that even in the absence of loaded drugs, ND-ODA also exerts a particular anti-inflammatory effect (Pentecost et al., 2017). Hydroxyapatite (HAP) is the main inorganic component of vertebrate bones and teeth, with excellent biocompatibility, bioactivity, and safety profile. Moreover, HAP is easily hydrolyzed under acidic conditions, enabling the use of nano-sized hydroxyapatite as a carrier for drug delivery (Ramesh et al., 2018). Pandey et al. (2021) incorporated MTX and teriflunomide into HA-coated HAP NPs for the treatment of RA. Modified HA can improve the encapsulation efficiency, hydrophilicity, and macrophage targeting of NPs. More importantly, HA can prolong the residence time and improve the timeliness of the drug because of its viscoelastic properties. As expected, the dual drug–loaded inorganic NPs can inhibit pro-inflammatory cytokines (TNF-α, IL-1β, and IL-6) and increase osteoblast and chondrocyte activity. In addition to inorganic material, researchers also use organic material nanocarriers to deliver drugs into the joint cavity for the manipulation of macrophages in the treatment of RA. Yeo et al. synthesized NO-scavenging polymer nano-gels for the efficient transdermal delivery of a NO-cleavable cross-linker, which reduces inflammation by scavenging NO *in vivo* (Park et al., 2017; Yeo et al., 2019). Jansen et al. (2019) also used organic nanomaterials to deliver therapeutic drugs through intra-articular injection. However, unlike other researchers, they targeted TNF-siRNA delivery, which results in the silencing of TNF in macrophages for the treatment of RA. Clodronate (CLO) is a first-generation bisphosphonate often used in patients with bone loss. It can inhibit the synthesis of inflammatory mediators and cytokines and induce apoptosis in macrophages. Russo et al. (2016) encapsulated CLO into chitosan NPs to prepare chitosan-CLO NPs, which were subsequently introduced into a poloxamer gel. Because of the targeting and retention in the inflamed region, the therapeutic effect of chitosan-CLO gel was markedly enhanced compared with that of pure CLO.

### Manipulation of Macrophages by Nanomaterials Administered Through Intraperitoneal Injection

Owing to its easiness and rapidity, intraperitoneal injection is a widely used administration method in experimental animal research. Due to the large area of the peritoneal cavity, dense blood vessels and lymphatics have a strong absorption capacity, promoting the absorption of liquids. Thus, considering the damage caused to the abdominal blood vessels, intraperitoneal injection can lead to cumulative irritation; thus, it is rarely used in clinical practice. Occasionally, it can be used for special treatments in animal experiments (Yahyaei et al., 2019). As one of the traditional treatments for arthritis, Au reagents have valuable properties (e.g., biocompatibility and superficial modification), which inhibit the infiltration of mononuclear macrophages into synovial tissue. Moreover, Au can selectively accumulate in inflammatory synovial tissues and form Au-rich sediments. Therefore, scholars have suggested that macrophages can be an essential target for Au reagents. On account of their direct anti-arthritic effects, Au clusters can be efficiently used as a nanodrug even without drug loading (Khan and Khan, 2018; Yuan et al., 2019b). The strong positive charge of the Sv peptide can assist Au clusters in penetrating the cell membrane. Therefore, Yuan et al. anchored the Au clusters with the Sv peptide *via* strong Au–S bonds in aqueous solution under mild conditions to prepare Au25Sv9. It was found that Au25Sv9 selectively gathered in synovial tissues of animals with arthritis after intraperitoneal injection. This effect inhibited the receptor activator of RANKL and indirectly reduced the generation of osteoclasts and bone erosion. Of note, Au25Sv9 can effectively inhibit the activation of NF-κB induced by RANKL, directly inhibit inflammation-induced differentiation of osteoclasts, and block inflammatory bone destruction (Yuan et al., 2019a). The same research group also evaluated the effect of GSH as the template for ultrasmall Au nanoclusters to manipulate macrophages in the treatment of RA. The GSH molecules combine with these 29 Au atoms through the Au–S bond to form Au_29_GSH_27_. GSH, as a natural peptide with a small hydrodynamic diameter, is a suitable surface ligand of the Au cluster to improve pharmacokinetic characteristics *in vivo* (Gao et al., 2019). Jain et al. (2015) used peritoneal macrophages as drug-loaded carriers, acting as Trojan horse vectors to transport IL-10 plasmid DNA to the inflamed joint. The surface of the nanocarrier was modified with the four-amino acid peptide tuftsin, which promotes phagocytosis by binding with Fc and neuropilin-1 receptors on macrophages. As the peritoneal macrophages migrate to the site of arthritis, the drug-loaded NPs are also transported to promote the polarization of M1 macrophages to M2 macrophages, thereby reducing inflammation.

### Manipulation of Macrophages by Nanomaterials Administered Orally

Chloroquine (CQ), an established antimalarial drug, exerts a specific immunoregulatory effect. Recently, CQ was used to treat immune-related diseases, such as RA, systemic lupus erythematosus, and solar erythema, and is thought to inhibit the expression of TNF-α in synovial tissue. Researchers encapsulated CQ into SLN, which was uptaken by the intestinal lymphatic region. Bhalekar et al. validated that the levels of TNF-α at the site of inflammation were reduced following the oral administration of CQ-SLN. In this study, they mixed Compritol 888 ATO with CQ to obtain a precise drug–lipid mixture and added span80 to prepare CQ-loaded SLN (CQ-SLN). It was found that an increase in span80 led to an increase in the particle size and entrapment efficiency. NPs enter the systemic circulation through clathrin- and caveolae-mediated endocytosis in the intestinal lymphatic region, specifically accumulating in the arthritis joint and reducing the progression of the disease (Bhalekar et al., 2016). However, few studies have investigated the manipulation of arthritis synovial macrophages through oral drug-loaded NPs for the treatment of RA. The new delivery strategy based on the lymphatic uptake of drug-loaded NPs through oral administration provides novel insights and directions for researchers and clinicians.

### Manipulation of Macrophages by Nanomaterials for the Diagnosis of RA

In patients with RA, the pathological damage to the structure of the joint cannot be reversed. Hence, early diagnosis and treatment of RA are extremely important, as they can delay and prevent its development and reduce damage to the joint. Recently, nanomaterials were used to manipulate macrophages for the early diagnosis of RA. The protein fibrin is a natural matrix for cell attachment, proliferation, and extracellular matrix formation during wound healing and bone formation. More importantly, fibrin NPs exhibit good biocompatibility, immunocompatibility, hemocompatibility, and biodegradability (Weisel and Litvinov, 2017). Periyathambi et al. used goat blood as a precursor to prepare fibrin. The fibrin was dissolved in NaOH, and the iron solution was added under vigorous stirring to form magnetic fibrin NPs (MFNPs). Subsequently, FA and MFNPs were combined to prepare FA-MFNPs by ethyl-3-(3-dimethylaminopropyl)carbodiimide/N-hydroxysuccinimide reaction. FA was modified to target FR-β on the membrane of macrophages. The results suggested that FA-MFNPs can be used as magnetic resonance imaging contrast agents to detect activated macrophages in the synovial tissue of joints, indicating early RA (Periyathambi et al., 2017). However, the researchers used goat-derived fibrin, which may not have immunogenicity. Thus, further experimental research is warranted to determine the usefulness of fibrin in this setting.

## Effects of Macrophage Manipulation by Nanomaterials

### Inflammatory Cytokines

In the pathogenesis of RA, a complex network is formed by the mutual regulation of various cytokines. Cytokines are small molecular proteins that act as important mediators of intercellular communication. They play a crucial role in response to various stimuli throughout the inflammatory process. The imbalance between pro- and anti-inflammatory cytokines is deemed responsible for the development of RA. Both the overexpression of pro-inflammatory cytokines and the insufficient formation of anti-inflammatory cytokines can easily lead to RA (Mateen et al., 2016; Chen Z. et al., 2019). Pro-inflammatory cytokines (IL-1, IL-6, IL-17, IL-8, IL-1β, etc.) are the main factors linked to RA. Thus, the inhibition of pro-inflammatory cytokines has been suggested as the primary approach to treating RA (Alam et al., 2017). GCs (PD and Dex), antirheumatic drugs (MTX), Chinese medicine monomers (CEL, TPT, and BAC), SOD, tacrolimus, CLO, QCT, CQ, and the NO-cleavable cross-linker are delivered by carrier nanomaterials for the manipulation of macrophages to reduce pro-inflammatory cytokines.

### Macrophage Polarization

Macrophages are a group of highly heterogeneous cells. According to their polarization state, macrophages are divided into M1 macrophages and M2 macrophages. Macrophages are highly plastic, and their polarization is affected by a variety of cytokines in the microenvironment. The activated macrophages are termed M1 macrophages, which can induce T helper 1 cell activation, promote inflammation, and accelerate the elimination of intracellular pathogens. Uncontrolled over-activation of M1 macrophages will cause excessive inflammation and tissue damage (Sun et al., 2017; Tardito et al., 2019). Macrophages can be polarized into M2 macrophages by T helper 2 cell cytokines, such as IL-4 and IL-13. In contrast, as anti-inflammatory factors, M2 macrophages inhibit inflammation, thereby promoting tissue repair (Quero et al., 2017; Funes et al., 2018). Therefore, the induction of M1 macrophages to M2 macrophages has gradually attracted attention in the treatment of RA. Ag+, NF-κB p65 siRNA, MTX, RES, and IL-10 plasmid DNA can be delivered to induce polarization of M1 macrophages into M2 macrophages for the treatment of RA.

### Macrophage Apoptosis

Apoptosis refers to the autonomous and orderly death of cells controlled by genes to maintain the stability of the internal environment, which is of great significance to multicellular organisms. In biological development, apoptosis enables the elimination of harmful cells (e.g., neoplastic and senescent cells) and ensures the development of tissues and the balance of the internal environment (Pistritto et al., 2016; Yang et al., 2020). Lack of apoptosis may lead to increased numbers of macrophages in RA. Therefore, inhibiting the activation and promoting the apoptosis of macrophages may be an effective method for the treatment of RA (Henc et al., 2017; Yang et al., 2021). The delivery of Mcl-1 siRNA, Cel, and MTX discussed in this review can inhibit the activation of macrophages, induce macrophage apoptosis, and indirectly result in a superior anti-arthritic activity.

## Biosafety of Nanomaterials

A variety of nanomaterial-based nanocarriers have been used for the macrophage manipulation in the treatment of RA. Typical nanocarriers were constructed using the inorganic materials, organic materials, or endogenous biomimetic materials which possess different sizes, structures, and surface characteristics. These nanocarriers can promote the specific accumulation of drugs in the inflammation site to enhance the anti-RA effects of different drugs. Recently, the biosafety of nanomaterials has attracted much attention from pharmacologists as an important impact factor of nanomedicine, which requires that the nanosystem possess high macrophage selectivity in the inflammatory location of RA with low toxicity. By far, there are no approved assessment criteria on the biosafety of nanomaterials. The evaluated biosafety results of nanomaterials are always obtained based on the *in vitro* or *in vivo* animal study; however, it is difficult to infer the security of nanomaterials in the body (Su et al., 2018).

Size is the most important parameter in toxicity evaluation of nanocarriers. Compared with large-size nanoparticles, nanoparticles with a smaller size might possess lower security, because of which they can easily penetrate the skin and reach various organs such as the lungs and the brain. Other factors such as the shape and surface charge of nanocarriers and the route of administration also affect the biosafety of nanomaterials (Bianco et al., 2017). For example, positively charged or cationic nanocarriers possess greater toxicity than other nanocarriers, and intravenous administration of nanocarriers has more side effects than oral administration.

Nanomaterials produced from biological materials always have no obvious toxicity for the body; however, it is also not easy to eliminate from the body when it acts as a nanocarrier because of the extensive tissue distribution, which therefore might result in *in vivo* toxicity (Zielińska et al., 2020). Most of the inorganic or organic nanocarriers (such as liposomes, micelles, dendrimers, mesoporous silica nanoparticles, gold nanocarriers, super paramagnetic iron oxide nanoparticles, etc.) are obtained by chemical synthesis. Thus, their cell compatibility, blood compatibility, and good immune compatibility remain to be further investigated. Interestingly, the endogenous biomimetic nanomaterials possess excellent biocompatibility, which can greatly reduce the immunogenicity of the nanocarriers, thus improving the biological safety of the produced nanomedicine (Hossen et al., 2019).

By far, the biosafety evaluation of nanocarriers is still a challenge that needs to be further explored. In addition, the technology of the extraction and separation of cell membranes or membrane-like structures to obtain the endogenous biomimetic nanomaterials still needs to be verified in clinical settings. Together, the endogenous biomimetic nanomaterials possess higher biological security than other nanomaterials and might act as the ideal nanocarrier with drug encapsulation to manipulate macrophages for RA treatment.

## Conclusion

The pathogenesis of RA is complicated and, currently, there is no treatment that can completely cure the disease. NSAIDs, DMARDs, GCs, biological agents, and other treatments can only relieve pain, prevent disease relapse, reduce articular damage, and improve physical function and quality of life. Nanomaterials have been widely used in the treatment of RA owing to their ability to improve the bioavailability of drugs and promote clinical efficacy. In recent years, studies have found that the abnormal metabolism of macrophages is involved in the pathogenesis and development of RA. Therefore, the use of nanomaterials to target synovial macrophages, induce macrophage polarization and apoptosis, inhibit the production of pro-inflammatory cytokines, and regulate the function of macrophages to treat autoimmune diseases (e.g., RA) has become a research hot spot. Although targeted agents have achieved success in the treatment of RA, various targeted carriers are characterized by multiple limitations, such as minor drug loading, poor stability, immature preparation techniques, lack of pharmacokinetic models, immature quality of evaluation indexes, insufficient effectiveness, and a poor safety profile. The distribution of carriers in non-targeted tissues may lead to toxicity. Surface PEGylation of nanomedicines can avoid recognition by the RES, thereby extending their circulation time in the blood and increasing their accumulation in inflammatory sites. However, it is terrible for the infiltration of nanomedicine in tissues, internalization, and lysosome escape. Most procedures for the preparation of nanomedicines are complicated, and the clinical cost is high. Although exogenous biomaterials are nontoxic to the human body, they may also lead to immune rejection. Future research should focus on overcoming these shortcomings. This article mainly presents the progress achieved in the research on nanomaterials targeting macrophages for the treatment of RA, including the role of macrophages, the shortcomings of current drugs and the advantages of nanomedicines, and the methods and effects of the manipulation of macrophages by nanomaterials. This review provides new directions for the targeting of macrophages in the treatment of RA.
